# Supplementary material for: A Glycoprotein-Based Surface-Enhanced Raman Spectroscopy–Lateral Flow Assay Method for Abrin and Ricin Detection
Source: Toxins (Basel). 2024 Jul 11;16(7):312. doi: 10.3390/toxins16070312 (PMC11280971; doi:10.3390/toxins16070312)
Supplement: Supplementary file 1 [file toxins-16-00312-s001.zip › toxins-2999368-supplementary.pdf]

Supplementary Information

A Glycoprotein-Based Surface-Enhanced Raman Spectroscopy–Lateral Flow Assay Method for Abrin and Ricin Detection

Lan Xiao [1,2], Li Luo[2,3], Jia Liu[2,4], Luyao Liu[1,2], Han Han[5], Rui Xiao[5], Lei Guo[2,*], Jianwei Xie[2], and Li Tang[1,*]

1 Key Laboratory of Ethnomedicine (Minzu University of China), Ministry of Education, School of Pharmacy, Minzu University of China, Beijing 100081, China; xlzcfhl16@163.com (L.X.)

2 Laboratory of Toxicant Analysis, Academy of Military Medical Sciences, and State Key Laboratory of Toxicology and Medical Countermeasures, Beijing 100850, China

3 Guangdong Lifotronic Biomedical Technology Co., Ltd., Dongguan 523808, China

4 College of Pharmacy, Hebei Science and Technology University, Shijiazhuang 050018, China

5 State Key Laboratory of Pathogen and Biosecurity, Academy of Military Medical Sciences, Beijing 100071, China

* Correspondence: tangli6972@muc.edu.cn (L.T.); guolei@bmi.ac.cn (L.G.); Tel.: +86-10-66931650(L.G.); +86-13910666972(L.T.)

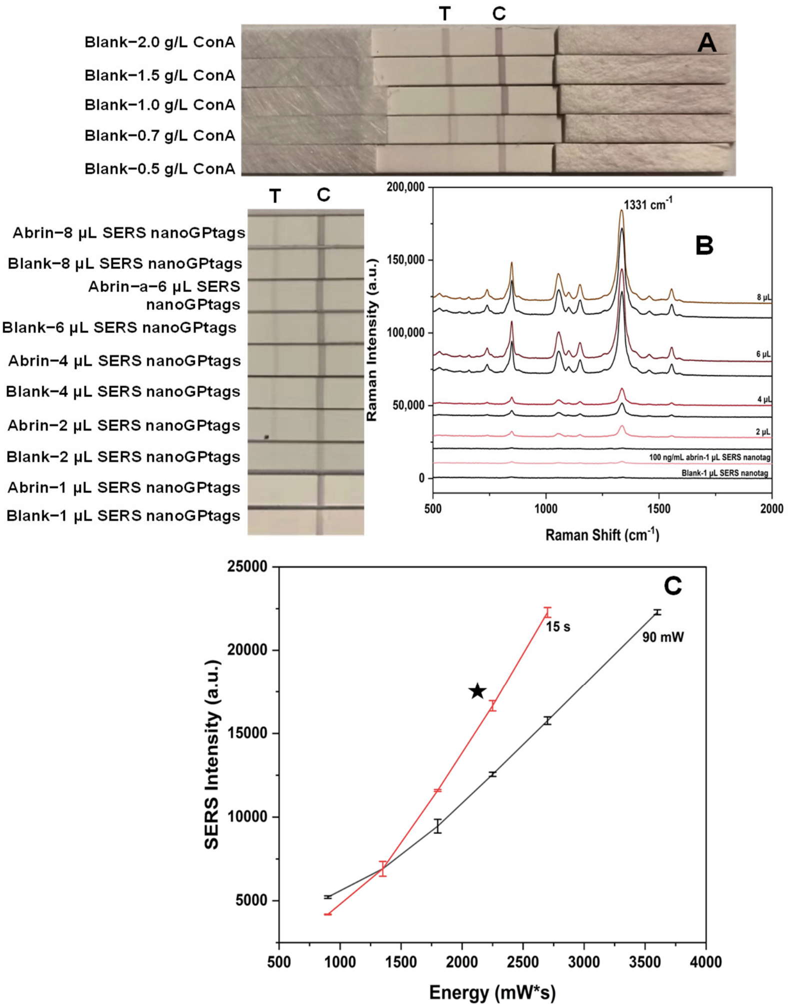

Figure S1. Optimization of experimental conditions for SERS–LFA strips prepared by the wet method; A: Optimization of concentration for the T line; B: Optimization of the optimized amount of SERS nanoGPtags; Optimization of integration time and laser power.

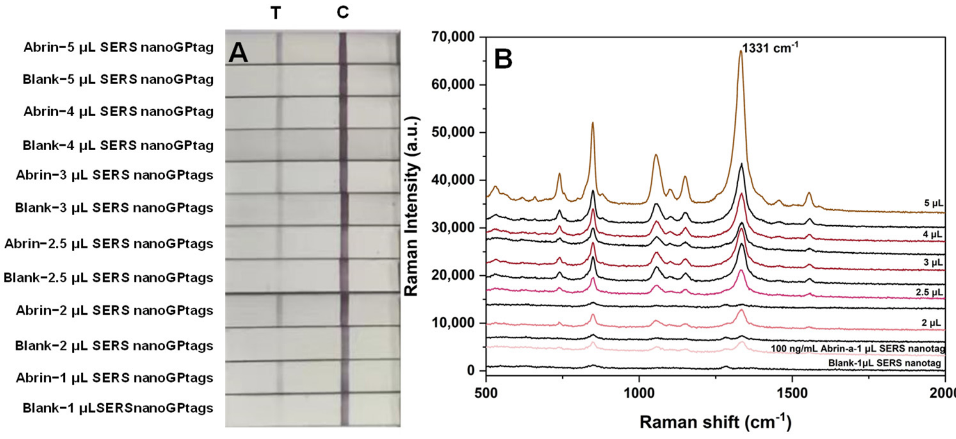

Figure S2. Optimization of SERS nanoGPtags in SERS–LFA strips prepared by the dry method.

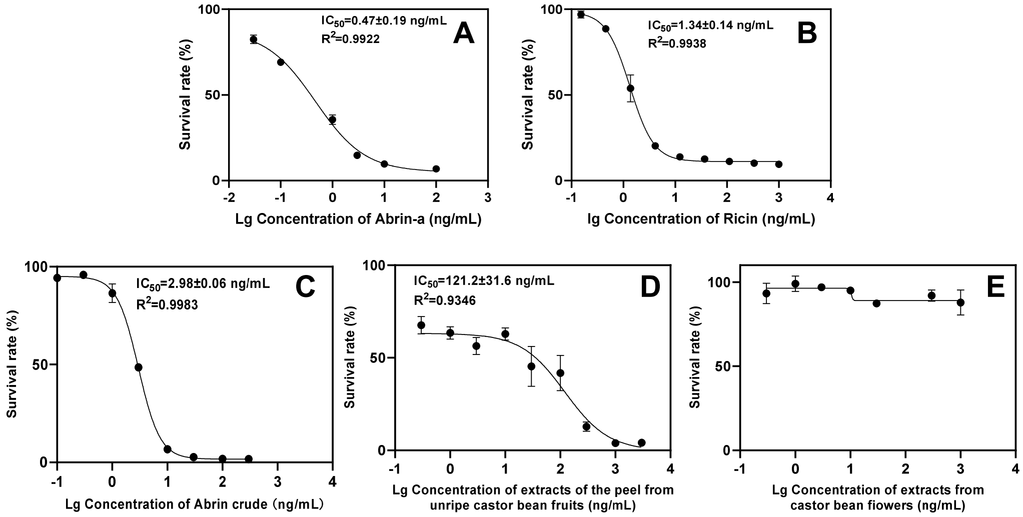

Figure S3. Cytotoxicity of abrin, ricin and real samples.

A: abrin; B: ricin; C: Abrin crude; D:extracts of the peel from unripe castor bean fruits; E: extracts from castor bean flowers.

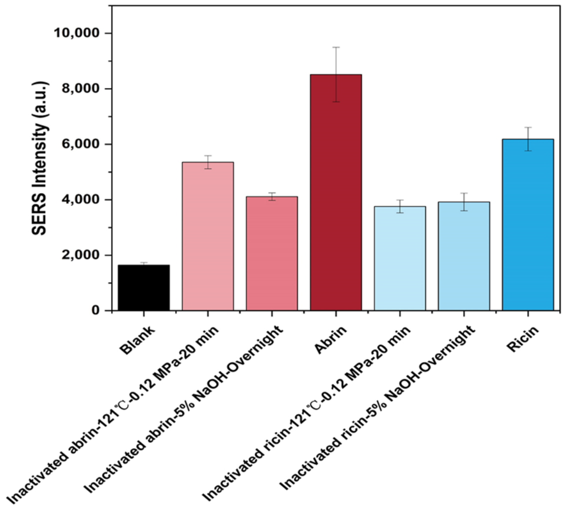

Figure S4. SERS–LFA detection of inactivated toxins under different conditions.

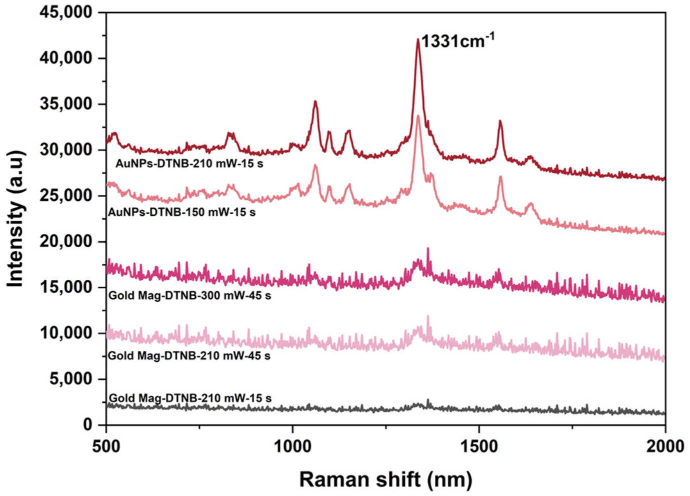

Figure S5. SERS spectra measured for AuNPs and Gold Mag.
